# Ideational factors associated with consistent use of insecticide-treated nets: a multi-country, multilevel analysis

**DOI:** 10.1186/s12936-022-04384-3

**Published:** 2022-12-06

**Authors:** Stella Babalola, Kuor Kumoji, Grace N. Awantang, Olamide A. Oyenubi, Michael Toso, Samantha Tsang, Therese Bleu, Dorothy Achu, Judith Hedge, David C. Schnabel, Shelby Cash, Lynn M. Van Lith, Anna C. McCartney-Melstad, Yannick Nkomou, Abdul Dosso, Wani Lahai, Gabrielle C. Hunter

**Affiliations:** 1 PMI Breakthrough ACTION Project, Johns Hopkins Center for Communication Programs, Baltimore, USA; 2 Department of Internal Medicine, Montefiore New Rochelle Hospital, New Rochelle, USA; 3 Department of Health, Behavior, and Society, Johns Hopkins Bloomberg School of Public Health, Baltimore, USA; 4 National Malaria Control Programme, Abidjan, Côte d’Ivoire; 5 National Malaria Control Programme, Yaounde, Cameroon; 6 U.S. President’s Malaria Initiative, Centers for Disease Control and Prevention, Yaounde, Cameroon; 7 U.S. President’s Malaria Initiative, Centers for Disease Control and Prevention, Freetown, Sierra Leone; 8 U.S. President’s Malaria Initiative, Centers for Disease Control and Prevention, Atlanta, USA; 9 PMI Breakthrough ACTION Project, Johns Hopkins Center for Communication Programs, Yaounde, Cameroon; 10 PMI Breakthrough ACTION Project, Johns Hopkins Center for Communication Programs, Abidjan, Côte d’Ivoire; 11 National Malaria Control Programme, Freetown, Sierra Leone

**Keywords:** ITN, Consistent use, Ideational variables, Multilevel, SBC

## Abstract

**Background:**

Malaria remains a major cause of morbidity and mortality in sub-Saharan Africa. Using insecticide-treated nets (ITNs) every night, year-round is critical to maximize protection against malaria. This study describes sociodemographic, psychosocial, and household factors associated with consistent ITN use in Cameroon, Côte d’Ivoire and Sierra Leone.

**Methods:**

Cross-sectional household surveys employed similar sampling procedures, data collection tools, and methods in three countries. The survey sample was nationally representative in Côte d’Ivoire, representative of the North and Far North regions in Cameroon, and representative of Bo and Port Loko districts in Sierra Leone. Analysis used multilevel logistic regression and sociodemographic, ideational, and household independent variables among households with at least one ITN to identify correlates of consistent ITN use, defined as sleeping under an ITN every night the preceding week.

**Findings:**

Consistent ITN use in Côte d’Ivoire was 65.4%, 72.6% in Cameroon, and 77.1% in Sierra Leone. While several sociodemographic and ideational variables were correlated with consistent ITN use, these varied across countries. Multilevel logistic regression results showed perceived self-efficacy to use ITNs and positive attitudes towards ITN use were variables associated with consistent use in all three countries. The perception of ITN use as a community norm was positively linked with consistent use in Cameroon and Côte d’Ivoire but was not significant in Sierra Leone. Perceived vulnerability to malaria was positively linked with consistent use in Cameroon and Sierra Leone but negatively correlated with the outcome in Côte d’Ivoire. Household net sufficiency was strongly and positively associated with consistent use in all three countries. Finally, the findings revealed strong clustering at the household and enumeration area (EA) levels, suggesting similarities in net use among respondents of the same EA and in the same household.

**Conclusions:**

There are similarities and differences in the variables associated with consistent ITN use across the three countries and several ideational variables are significant. The findings suggest that a social and behaviour change strategy based on the ideation model is relevant for increasing consistent ITN use and can inform specific strategies for each context. Finally, ensuring household net sufficiency is essential.

**Supplementary Information:**

The online version contains supplementary material available at 10.1186/s12936-022-04384-3.

## Background

Increased international attention and deployment of effective malaria prevention tools have achieved significant reductions in malaria’s health impact over the past 20 years. There is strong evidence that insecticide-treated nets (ITNs) are an effective method of malaria prevention by killing mosquito vectors, creating a physical barrier, and acting as a repellent [1–3]. In sub-Saharan Africa, where the majority of malaria infections are spread by *P. falciparum,* use of ITNs has contributed the most to decreases in clinical cases averted [4]. A combination of increased access and high rates of consistent ITN use are necessary to achieve reductions in malaria-related morbidity and mortality [5].

From a supply perspective, widespread adoption of the World Health Organization’s (WHO) recommendation for universal ITN coverage (one ITN for every two persons in all households) has increased availability of ITNs throughout sub-Saharan Africa. From the demand perspective, nationally representative data from countries throughout sub-Saharan Africa show high levels of ITN use among those with access to an ITN [6]. However, despite this progress, just half of those at risk of malaria in sub-Saharan Africa slept under an ITN the night before being surveyed [7]. As efforts to increase access to ITNs intensify, programme planners also must learn more about how to achieve higher rates of consistent net use.

Malaria is a leading cause of morbidity and mortality in the countries included in the present study: Cameroon, Côte d’Ivoire, and Sierra Leone [7]. According to each country's Malaria Operational Plan, while malaria transmission is stratified and seasonal in all three countries, these variances are more pronounced in Cameroon where transmission is less intense and more seasonal in the North and Far North regions [8]. All three countries have adopted the WHO recommendation of universal ITN coverage, achieving varying degrees of access and use [8, 9, 10].

In 2018, less than two thirds (59%) of Cameroonians had access to an ITN, defined as the percentage of the de facto household population, who could sleep under an ITN if each ITN in the household was used by up to two people [11]. In 2016, about half (47%) of Ivoirians had access [12] and in 2019 a quarter of Sierra Leoneans had access to an ITN [13]. Recent data on the ITN use:access ratio (calculated as the proportion of the population that used an ITN the previous night divided by the proportion of the population with access to an ITN within their household) indicate a high use-access ratio in the three countries: Cameroon (84%; 2014), Côte d’Ivoire (79%; 2016), and Sierra Leone (98%; 2017) [6]. It is however pertinent to note that this indicator varies significantly within countries [6].

Access to an ITN is a key determinant of use and national surveys across sub-Saharan Africa demonstrate that the majority of individuals with enough ITNs in their household (that is, with at least one net per two people) use them [6, 14, 15]. All the same, a gap remains between having enough nets and use of those nets [16]. For example, in a 29-country study [17], Olapeju et al. found that whereas differences in ITN use across demographic groups were attenuated in households with enough nets compared to households with not enough nets, in the former households, some groups were still more or less likely than others to use nets. There is increasing recognition of the role of sociodemographic, ideational (psychosocial), and household factors as significant predictors of ITN use and several studies have examined some of these factors.

In terms of demographics, ITN use tends to be more common among young children than older children or adults [17–22], more common among younger women than older women [23], and more common among adult women (particularly pregnant women) than adult men [17, 24–26]. Findings regarding a mother’s educational level and ITN use are mixed [27–31]. There is mixed evidence on ITN use among those in urban and rural households [29, 32–34]. Age and sex differentials in ITN use may be moderated by household net coverage such that differences in ITN use between men and children under the age of five years are reduced when the analysis is limited to households with enough nets [17]. Studies that have examined the correlation between educational attainment and ITN use have yielded mixed results [27–34]. Evidence of the association between socioeconomic status and ITN use is also mixed [17, 27, 28, 33, 35–37]. Several studies found that the odds of ITN use were lower in larger households, especially those with young children, than in smaller households [28, 38, 39]. There is evidence of a positive link between the number of sleeping spaces and ITN use [25, 40–42].

There is evidence that exposure to social and behaviour change interventions has led to increased net use in many African countries. Studies have also found that exposure to malaria-related information from the media or other sources significantly increases the likelihood of ITN use [43–47].

An increasing body of literature has focused on the association between ideational factors and behaviors related to malaria like care seeking for fever [48, 49], uptake of intermittent preventive therapy during pregnancy [50, 51] and the use of ITNs [32, 39, 46, 52]. Several recent studies indicate that knowledge about the benefits of ITNs is an important correlate of use in low and high transmission areas [24, 39, 41, 53]. Studies in many countries in Africa have revealed an association between perceived self-efficacy and ITN use [32, 39, 54, 55]. Other studies suggest that perceived social support and interpersonal communication can also be important determinants of ITN use [31, 32, 45, 56]. Descriptive norm, or a person’s perception about the net use behavior of people in their community, has been found to be positively associated with the use of ITNs [32, 39, 57]. Positive attitudes toward ITNs are also associated with higher rates of ITN use [32, 58].

Knowledge about where to purchase an ITN and willingness to buy one were also correlated with higher rates of use [32]. Further, trust in a net’s efficacy, or a person’s perceived response-efficacy, has been correlated with higher consistent net use [32, 39]. Evidence related to the correlation between perceived severity of malaria and ITN use is mixed [39, 46, 55, 59].

As the foregoing review reveals, a growing body of research describes sociodemographic, ideational, and household predictors of ITN use. However, these studies have generally focused on net use the previous night [29, 39, 46]. More data is needed to understand what drives household members to maintain consistent use of ITNs over time. Moreover, whereas several single-country studies have examined correlates of consistent use [25, 32, 60], this multi-country analysis broadens the line of inquiry, using a multilevel analytic approach to describe variations in consistent use of ITN among men and women of reproductive age in three countries in Western and Central Africa: Cameroon, Côte d’Ivoire, and Sierra Leone. The Malaria Behavior Survey (MBS) is a comprehensive survey on malaria-related behaviours and their drivers. The MBS was first implemented in these three countries and, while differing slightly in sampling approaches, provides an opportunity for a broader analysis of net use behaviours. A multi-country focus was determined to be potentially more useful to the broader malaria community as findings implicate a larger population across the region. The results allow us to identify the ideational variables significantly associated with consistent net use in the three countries, with the ultimate purpose of informing malaria behaviour change programmes.

## Methods

The data analysed were derived from the first three MBSs conducted in Cameroon [8], Côte d’Ivoire [9], and Sierra Leone [10]. The MBS is a household survey informed by the ideation model [61] of behaviour change and designed to assess ideational and other factors associated with malaria-related behaviours using a standardized methodology as described at https://malariabehaviorsurvey.org [62]. The sample was nationally representative in Côte d’Ivoire, representative of two districts (Bo and Port Loko) in Sierra Leone, and in two regions (North and Far North) in Cameroon. In Cameroon, data collection overlapped with mass net distribution in the North region and preceded the activity by a few weeks in the Far North. In Côte d’Ivoire, data collection occurred about nine months after the most recent mass net distribution in 2017 while in Sierra Leone, the survey took place about six months before the 2020 mass net distribution.

The sampling procedures were similar in the three countries based on selection of enumeration areas (EA) and households (about 20 per EA, on average); within selected households all women of reproductive age were invited to respond to the survey and one man in one-third of the selected households. The survey questions were also similar across countries. The total sample size was 4514 (949 men and 3565 women) in Cameroon, 8566 (1846 men and 6720 women) in Côte d’Ivoire, and 3836 (627 men and 3209 women) in Sierra Leone, and. The analyses presented here, however, only focus on respondents from households with at least one ITN: 2995 in Cameroon, 6164 in Côte d’Ivoire, and 2730 in Sierra Leone. During the survey, respondents were asked how many nights, on average, they slept under an ITN in a week. The dependent variable is consistent use of an ITN, defined as sleeping under an ITN every night of the week. In the study countries, a huge proportion (98% or more) of the enumerated bed nets in the households were ITNs; therefore, in this manuscript all types of nets in the household are described as ITNs. The independent variables are described in Table 1 and include a range of sociodemographic and ideational factors identified by the ideation model as important for behaviour change. Multicollinearity was assessed by generating a dependent normally distributed random variable and using the ideational variables as independent variables to estimate this random variable in a linear regression model. The variance inflation factor (VIF) from this linear regression model served to assess multicollinearity among the ideational variables. The results, presented in Additional file 1: Table S1, show that multicollinearity was not an issue in any of the three study countries. The VIF for the individual ideational variables as well as the mean VIF were generally under 2.0.

**Table 1:** Independent variables included in the estimated multilevel models

Respondent’s sex	Male or female
Respondent’s current age	Measured in single years
Respondent’s level of education	Defined as none, primary, and secondary education or higher
Perceived severity of malaria	Based on four items that assessed the perceived severity of malaria (Additional file 1: Table S1). Responses that reflect the perception that malaria is a serious condition were scored + 1, those that reflect lack of perceived severity were scored -1 while neutral responses were scored 0. The scores for all items were combined, and the total score split at 0 to denote perceived versus non-perceived severity
Perceived susceptibility to malaria	Based on four items that assessed the extent to which the respondents perceived themselves to be at risk for malaria (Additional file 1: Table S1). The items were scored, combined and split at 0
Attitudes towards bed nets	Derived from six items measured on the Likert scale (Additional file 1: Table S1). The items were scored, combined, and split at the median
Perceived response-efficacy of nets	Derived from three items that assessed beliefs about the efficacy of bed nets (Additional file 1: Table S1). The items were scored, combined, and split at 0
Perceived self-efficacy for net use	Derived from four items that assessed the respondent’s level of confidence in their ability to use nets for themselves or their children (Additional file 1: Table S1). The items were scored, combined, and split at the median
Descriptive norm about net use	Derived from a question that asked what proportion of people in the respondent’s community sleeps under a net each night. Response denoting at least half of community members sleep under a net were scored 1 while other responses were scored 0
Interpersonal communication about malaria	Discussion of malaria with others, including spouse, friends and family
Exposure to malaria-related messages	Exposure to malaria messages from the media and community sources
Integrity of the ceiling of the dwelling unit	Defined as completely sealed versus partially sealed or absent ceiling
Household wealth quintile	Derived from selected household assets and dwelling characteristics using principal component analysis
Presence of under-five children in the household	Variable derived from the household schedule
Household net coverage	Defined as whether the household has at least one ITN for every two members
Type of place of residence	Defined as urban versus rural residence
Region/zone/district of residence	Defined as study regions in Cameroon, study zones in Côte d’Ivoire, and study districts in Sierra Leone

The main analytic method was multilevel logistic regression that accounts for clustering of behaviour at the household and EA (cluster) level. Two models were estimated for each country: (1) an empty model that assesses if there is enough clustering to warrant the use of a multilevel model as this result would indicate that there are variables operating at the cluster level that influence the use of nets, and (2) a full model that assesses random effects of the household and EA and fixed effects of the independent variables described in Table 1. The results of the estimated models (adjusted odds ratio and relevant random effects statistics) are presented in Table 2.

**Table 2:** Results (adjusted odds ratio, random effects) of multilevel logistic models of consistent ITN use on selected socio-demographic, ideational, household and community variables; Cameroon, Côte d’Ivoire and Sierra Leone

Predictors	Cameroon		Côte d’Ivoire		Sierra Leone	
	Empty model	Full model	Empty model	Full model	Empty model	Full model
Sociodemographic characteristics and message exposure						
Sex	–		–		–	
Male (RC)		1.000		1.000		1.000
Female		3.642^d^		1.623^c^		1.258
Age in years	–	1.045^d^	–	1.031^d^	–	1.105^b^
Square of age		–		–		0.998^b^
Education level	–		–		–	
None (RC)		1.000		1.000		1.000
Primary		1.261		0.621^d^		0.568^c^
Secondary +		1.337		0.434^d^		0.589^c^
Exposure to malaria-related messages in past six months	–	0.586^b^	–	1.489^c^	–	1.598^c^
Ideational characteristics						
Positive attitudes towards net use	–	3.171^d^	–	7.593^d^	–	1.455^b^
Perceived severity of malaria	–	1.361	–	0.874	–	1.115
Perceived vulnerability to malaria	–	2.322^d^	–	0.731^b^	–	1.658^c^
Discussed malaria with others	–	0.874	–	2.209^d^	–	0.539^d^
Perceived response efficacy of ITNs	–	0.721	–	2.005^d^	–	2.013^d^
Perceived self-efficacy to use nets	–	7.026^d^	–	3.951^d^	–	3.246^d^
Perceived net use as a community norm	–	2.891^d^	–	1.319^b^	–	1.168
Household and community characteristics						
Household has at least one child under age 5	–	2.133^c^	–	2.035^d^	–	1.105
Household wealth quintile	–		–		–	
Lowest (RC)						1.000
Second		1.000		1.000		1.188
Middle		2.152^b^		0.612^b^		1.184
Fourth		4.050^c^		0.517^b^		0.935
Highest		6.519^d^		0.344^d^		0.934
Net supply within household	–		–		–	
Insufficient (RC)		1.000		1.000		
Sufficient		2.978^d^		3.044^d^		2.023^c^
Dwelling unit has completely sealed ceiling	–	0.972	–	0.578^c^	–	1.091
Urban residence (RC = rural)	–	0.532	–	0.921	–	0.459^a^
Region of residence	––	1.000				
Far North (RC)		0.067^d^				
North						
Zone of Residence			–			
North (RC)				1.000		
Central				1.332		
South				1.255		
Abidjan				0.086^d^		
District of residence					–	
Bo (RC)						1.000
Port Loko						0.347^c^
Random effects						
Household level variance (SE)	22.792 (6.696)	14.761 (3.142)	13.014 (1.8329)	11.047 (1.568)	1.833 (0.397)	2.114 (0.476)
Cluster level variance (SE)	3.419 (0.857)	1.971 (0.576)	2.909 (0.576)	0.344 (0.143)	3.488 (0.687)	2.537 (0.560)
LR Test (multilevel vs. logistic): ***Χ***^2^/p	541.9/0.000	393.2/0.000	890.5/0.000	501.2/0.000	645.9/0.000	354.0/0.000
Intraclass correlation (Household)	0.888	0.835	0.829	0.776	0.618	0.586
Intraclass correlation (Cluster)	0.116	0.098	0.151	0.023	0.405	0.319
Proportional change in variance (Household)	–	35.2%	–	15.1%	–	− 15.3%
Proportional change in variance (Cluster)		42.3%	–	88.2%		27.3%
Number of observations	2995		6164		2730	

*RC* reference category

^a^p < .1

^b^p < .05

^c^p < .01

^d^p < .001

## Results

There are commonalities and differences in the results across the study countries which are presented by country, then discussed across countries.

### Cameroon

#### Variations in consistent net use

Bivariate analysis results indicate that the proportion of individuals in households with at least one ITN who used nets consistently varied between the two regions and by sex, but not by other sociodemographic variables. Overall, more than two-thirds (72.6%) of the respondents reported consistent net use. The proportion of individuals who slept under a net consistently was significantly higher among female respondents (75.4%) than male respondents (69.2%): p < 0.001. Similarly, there was a significant difference between the regions: the North (64.4%) and the Far North (79.5%); p < 0.001. Consistent use of nets did not vary significantly by a respondent’s education level, place of residence, or household wealth quintile.

#### Multilevel analysis

The estimated intraclass correlation coefficients (ICC) in the empty model indicate significant clustering of consistent net use (Table 2) within EAs and, especially, within households in the same EA. The data show that 11.6% of the variance in consistent net use is attributable to clustering at the EA level while households within EA clustering contribute 88.8% of the variance. This result justifies the use of a multilevel model.

The full model adjusts the odds of sleeping under a net consistently based on individual and household determinants. The addition of these variables led to a 35.2% proportional change in variance at the household level and 42.3% at the EA level, indicating that a sizable proportion of the variance in the empty model was due to differences in the included determinants across households and EAs. After adjusting for the determinants, the results for the full model revealed that consistent net use was significantly and positively correlated with some sociodemographic variables, including the individual’s sex and age, presence of at least one child under five years old in the household, availability of at least one net for every two household members, and household wealth quintile. The ideational variables associated with higher odds of consistent net use were positive attitudes towards nets, perceived vulnerability to malaria, perceived self-efficacy to sleep under a net, and the belief that net use was a community norm. In contrast, exposure to malaria-related messages in the last six months and residence in the North rather than the Far North region were negatively associated with the outcome.

Specifically, women were more than three times as likely to sleep under a net every night compared to men (AOR = 3.642, p < 0.001). The model suggests that the odds of an individual’s consistent net use increase by 4.6% with each additional year of age (AOR = 1.046, p < 0.001). While there was no significant relationship between a respondent’s education level and the odds of them using a net consistently, men and women who were exposed to malaria messages were surprisingly 41% less likely to report consistent use compared to their peers not exposed (AOR = 0.586, p < 0.05).

In terms of ideational variables, having positive attitudes related to net use was associated with more than a three-fold increase in the odds of consistent net use (AOR = 3.171, p < 0.001) relative to not having such attitudes. Perceived vulnerability to malaria increased the odds of consistent use by 132% (AOR = 2.322, p < 0.001). Similarly, the odds of consistent use were higher if people were confident in their ability to use nets (AOR = 7.026, p < 0.000), or if they perceived net use as a norm in their community (AOR = 2.891, p < 0.001). On the other hand, the odds of consistent net use were not significantly associated with recent discussion about malaria, perceived severity of malaria, and the perception that nets effectively prevent malaria.

The results revealed the importance of some household variables. For example, individuals living in households with at least one net per two people had greater odds of using a net (AOR = 2.978, p < 0.001) relative to similar individuals in households without enough nets. The association with household wealth quintile was positive although not monotonically so. For instance, living in a household belonging to the fourth wealth quintile was associated with more than a six-fold increase in the odds of consistent net use (AOR = 6.519, p < 0.001) whereas living in a household belonging to the highest wealth quintile was associated with a three-fold increased odds (AOR = 3.095, p < 0.05) compared to the lowest quintile. The presence of a child aged less than five years old in the household increased the odds of consistent ITN use more than two-fold. Finally, living in the North region was associated with significantly decreased odds of consistent use compared to the Far North (AOR = 0.067, p < 0.001).

### Côte d’Ivoire

#### Variations in consistent net use

About two-thirds of the respondents in households with at least one ITN reported sleeping under an ITN every night. There were no differences by sex (men—65.3%; women—65.6%). There were, however, significant differences by survey district, urban residence, education level, and household wealth quintile. Consistent net use was less common in Abidjan (38.9%) than in other zones (74.1% in the North, 72.6% in the Center, and 72.2% in the South) The behaviour was also significantly less common in urban areas (57.3%) than in rural areas (76.6%). In addition, consistent net use decreased monotonically with level of education from 73.0% among the respondents with no formal education to 65.4% among their peers with primary education and 57.5% among those with secondary education or higher.

#### Multilevel analysis

The empty model (Table 2) reveals significant clustering at the EA and household levels. The intraclass correlation indicates that clustering at the EA level accounted for 15.1% of the total variance while households within EA clustering accounted for 82.9% of the variance. This result indicates that a significant portion of the variance in consistent net use is due to factors operating at the household and EA levels and justifies the use of multilevel modelling.

Conditional on the covariates in the full model (Table 2), there is hardly any more clustering at the EA level (2.3% of the residual variance) whereas clustering within the same household and EA remains strong (77.6% of the residual variance). The introduction of the correlates into the model resulted in a significant 88.2%% proportional change in EA-level variance, indicating that a major portion of the EA level clustering observed in the empty model is due to inter-cluster differences in the correlates included in the estimated model. The results further revealed significant association of consistent use of ITNs with sociodemographic, ideational, and community variables. The results show that women were 62% more likely than men to report consistent use of ITN (aOR-1.622; p < 0.001). The association with age was strong with a unit increase in age increasing the odds of consistent net use by 3.1% (AOR = 1.031; p < 0.001). There was a dose–response negative association with education level such that respondents with primary education were 38% less likely (AOR = 0.621; p < 0.001) and those with secondary education or higher were 57% less likely (AOR = 0.434; p < 0.001) to report consistent net use compared to their peers with no education. Exposure to malaria-related messages in the six months before the survey increased the odds of consistent net use by 49% (AOR = 1.489; p < 0.01).

Regarding ideational variables, the strongest correlates of consistent net use were positive attitudes towards net use, perceived vulnerability, interpersonal communication about malaria, perceived response-efficacy of nets, perceived self-efficacy for net use, and descriptive norm about net use. Positive attitudes towards net use increased the odds of consistent ITN use more than seven-fold (AOR = 7.593; p < 0.001). Perceived self-efficacy for net use was associated with almost a four-fold increase in the odds of consistent use (AOR = 3.591; p < 0.001) while interpersonal communication increased the odds by about 121% (AOR = 2.209; p < 0.001). The perception that net use was a community norm was associated with a 32% increase in the odds of consistent ITN use. Respondents who perceived that nets are effective for malaria prevention were twice as likely to report consistent net use compared to their peers that did not believe in the response-efficacy of nets. In contrast, perceived vulnerability decreased the odds by 27% (AOR = 0.731; p < 0.05).

The presence of a child under five years of age in the household increased the odds of consistent ITN use about two-fold (AOR = 2.035; p < 0.001) whereas an intact ceiling in the dwelling unit was associated with reduced odds of consistent net use (AOR = 0.578; p < 0.01). The association with household wealth was negative such that respondents in higher wealth quintiles were significantly less likely to report consistent net use compared with their peers in the lowest wealth quintile. Living in a household with adequate net coverage (that is, with at least one net for two persons) increased the odds of consistent net use about three-fold (AOR = 3.044; p < 0.001). Urban residence did not appear to make a conspicuous difference. There were clear variations by zone of residence although the differences were only significant when Abidjan was compared with the other zones. Compared to living in the North, residence in Abidjan was associated with 91% reduced odds of consistent net use. A test of differences among the odds associated with the zones revealed that the odds of consistent net use was significantly lower in Abidjan compared to the Central zone (p < 0.001) or the South (p < 0.001).

### Sierra Leone

#### Variations in consistent net use

Overall, more than three-quarters (77.1%) of respondents in households with at least one ITN reported that they had slept under an ITN every night of the previous week. Significant differences in net use were observed by place of residence, level of education, and household wealth quintile. A higher proportion of respondents in Bo compared to Port Loko (86.4% vs. 63.6%; p < 0.001) and rural areas compared to urban areas (81.0% vs. 64.7%; p < 0.001) reported that they had slept under an ITN every night of the previous week. The use of ITNs was inversely related to educational status. Fewer respondents with primary education (73.8%; p < 0.05), and higher education (73.5%; p < 0.05) reported consistent use of ITNs, compared to respondents with no education (81.0%). Similarly, consistent net use was significantly higher among respondents in the lowest wealth quintile (84.8%) compared to respondents in the fourth (73.8%; p < 0.01) and highest wealth quintile (71.6%; p < 0.01). No significant difference was observed between men (78.6%) and women (75.8%) in the proportion of individuals who reported consistent ITN use.

#### Multilevel analysis

There was significant clustering of consistent net use at the EA and household levels. The intraclass correlation (ICC) for the empty model indicated that clustering at the EA level accounted for 40.5% of the total variance while households within EA clustering accounted for 61.8% of the variance. These results show that a significant portion of the variance in consistent net use was due to factors operating at the household and EA levels and justifies the use of multilevel modelling for the analysis.

The full model (Table 2), which adjusts for individual and household factors, showed that after adding covariates to the model, clustering persisted at the EA level (31.9% of the residual variance) and also remained strong within the same household and EA (58.6% of the residual variance). Nonetheless, the full model showed a 27.3% proportional change in variance at the EA level, indicating that a moderate proportion of the EA level variance in the empty model was due to differences in the included determinants across EAs. In contrast, the household-level variance actually increased by 15.3%, indicating a negative correlation between one or more of the correlates and household-level errors [63]. A closer look at the Sierra Leone data shows that perceived self-efficacy is the variable mainly responsible for this unexpected finding.

The results showed significant associations between consistent ITN use and sociodemographic and ideation variables. There was a curvilinear relationship between age and consistent net use. Specifically, the odds of reporting consistent ITN use steadily until the age of 30 years and then declined. An inverse relationship was observed between consistent net use and education. Primary education (AOR = 0.568; p < 0.01) and higher education (AOR = 0.590; p < 0.01) were significantly associated with reduced odds of consistent net use compared to no education. Exposure to messages about malaria in the past six months resulted in about 60% increase in the odds of consistent net use (AOR = 1.598; p < 0.01).

Several ideational variables were associated with consistent ITN use. Perceived self-efficacy to use nets was the strongest predictor of consistent net use and was associated with a three-fold increase in the odds of consistent net use (AOR = 3.246; p < 0.001). Perceived efficacy of nets was associated with a two-fold increase in the odds of consistent net use (AOR = 2.013; p < 0.001), and perceived vulnerability to malaria was associated with 66% increase in the odds of consistent use of ITNs (AOR = 1.658; p < 0.01).

Living in a household with at least one net for every two persons was associated with a two-fold increase in the odds of consistent net use (AOR = 2.023; p < 0.01). Residence in Port Loko was associated with a 65% decrease in the odds of consistent ITN use compared to Bo (AOR = 0.347; p < 0.01) district. Presence of a child under the age of five years in the household, integrity of the ceiling of the dwelling unit, household wealth quintile, and urban residence were not significantly associated with consistent ITN use.

## Discussion

Using data from the first three Malaria Behaviour Surveys conducted in three African countries between 2018 and 2019, this manuscript assessed and compared the factors associated with consistent use of ITNs among households with at least one ITN. Analysis of these data from Côte d’Ivoire, two regions of Cameroon, and two districts of Sierra Leone explored whether similarities or differences exist that might inform and improve programmatic decisions about how to influence behaviours like consistent ITN use in these and similar settings. Consistent with the summary of extant literature described above, the results from Cameroon, Côte d’Ivoire, and Sierra Leone revealed commonalities in the correlates of consistent net use as well as noticeable differences.

In general, the results underscored the value of the ideation model for better understanding the determinants of malaria-related behaviours. As summarized in the following paragraphs, the findings echo what recent studies of the correlates of ITN use have demonstrated, while also providing new perspectives and insights regarding what should be the focus of SBC strategies for promoting consistent ITN use among households with at least one ITN in the study countries.

None of the socio-demographic variables showed a consistent pattern of association with the outcome across the three countries. For example, current age was positively associated with consistent use in Cameroon and Côte d’Ivoire, indicating a tendency for prioritizing older individuals in ITN allocations within the households. In Sierra Leone, the relationship was curvilinear with consistent use initially increasing with age until 30 years, and thereafter decreasing as age increased. Findings showing a positive relationship between age and use of mosquito nets among adults are rare in the literature. While Babalola et al. [32] found that the odds of consistent use of ITNs decreased with age in their three-state study in Nigeria, Idowu et al. [64] found a curvilinear relationship in rural Western Nigeria consistent with what this manuscript found in Sierra Leone. The relationship with respondent’s sex was strong in Cameroon and Côte d’Ivoire but insignificant in Sierra Leone. The finding for Cameroon and Côte d’Ivoire is consistent with findings from other studies in Africa [17, 24, 32]. In a secondary analysis based on Demographic and Health Surveys (DHS) and Malaria Indicator Surveys (MIS) data from 29 countries, Olapeju et al. [17] found that in most of the countries, women (pregnant or not) were more likely to sleep under a net compared to men. The same study also found no significant association between respondent’s sex and ITN use in a few countries, including Cameroon, Burundi, Congo-Brazzaville, and Liberia.

Higher levels of education were not significantly correlated with consistent net use in Cameroon but were negatively correlated with use in Côte d’Ivoire and Sierra Leone. Both patterns have been found in the literature. For example, Kanmiki et al. [30] found no significant relationship between education and net use in northern Ghana, while Russell et al. [31] found a negative relationship in south-eastern Nigeria. Considering that the estimated models controlled for other variables (e.g., urban residence) that are known to be associated with education and lower net use, the reasons for the negative association of consistent ITN use with education level are not clear. However, the negative association may be due to characteristics of the dwelling unit (presence of mosquito screening on windows and doors, air conditioning or fans) that give the sense of protection from mosquitoes and make people perceive the use of ITNs as redundant.

Consistent with what many other studies have found [31–33, 52] the relationship of exposure to malaria-related messaging with consistent net use was positive in Côte d’Ivoire and Sierra Leone. In Côte d’Ivoire, the SBC strategies that were implemented in the six months before the survey included interpersonal communication and community mobilization activities implemented by CHWs in 42 rural districts. In addition, television spots were disseminated nationally, and radio jingles were played on three faith-based radio stations. The media materials and the CHW activities addressed various malaria-related behaviours including use of ITNs. In Sierra Leone, in the 6 months prior to implementation of the MBS, a number of SBC activities were implemented to promote continued uptake of consistent ITN use. This included nationwide broadcasting of malaria jingles and approaches to identify and use positive deviant community volunteers to promote ITN use at the community level. In addition, in preparation for the 2020 mass net distribution campaign, there were a number of advocacy meetings with paramount chiefs to discuss the implementation of SBC activities during the 2020 mass campaign to identify key lessons learned and key technical SBC input and feedback in micro planning meetings. In Cameroon, the focus of the SBC efforts during the six months preceding the survey was threefold: (1) the mass net distribution campaign: explaining how to obtain nets and prepare the new nets for installation through community mobilizers, community radio and town criers; (2) the SMC campaign: using community mobilizers and community radio to explain the importance, process of administration and required dosage of the SMC drug; and (3) using community mobilizers and community radio to explain the importance of sleeping under an ITN every night, along with other malaria-related behaviours. The SBC activities in Côte d’Ivoire and Sierra Leone may be influencing desired behaviour change; expanding those activities may increase ITN use. In contrast, this study showed a surprisingly significant negative relationship of exposure to malaria-related messaging with consistent use in Cameroon. The reason for this unusual finding is not clear and warrants further investigation.

Consistent with evidence from previous studies [31, 32, 54, 55, 65], the current study found that ideational variables are important determinants of consistent use of ITNs (see summary on Table 3). Not all ideational variables were, however, significant across the three countries. Perceived self-efficacy to use ITNs and positive attitudes towards nets were the only ideational variables that demonstrated the same pattern of positive association with consistent use across the three countries, echoing findings from other studies [32, 54, 55]. The positive association with attitudes towards ITNs found in the three study countries is consistent with findings from previous studies [32, 58]. The association with perceived severity was not significant in any of the three countries. This finding is inconsistent with Watanabe et al. [59], Hung et al. [55] and Asingizwe et al. [54] who found a positive relationship in their studies in Vanuatu (in one of the two study islands), Taiwan, and Rwanda, respectively. Unlike Babalola et al. [32] who found no significant relationship between perceived susceptibility and consistent use of ITNs, the present study found a negative relationship in Côte d’Ivoire and a positive association in the other two countries.

**Table 3:** Summary of the association of consistent ITN use with specific ideational variables

Ideational variable	Cameroon	Côte d’Ivoire	Sierra Leone
Positive attitudes towards net use	+ ^c^	+ ^c^	+ ^a^
Perceived severity of malaria	+ ns	− ns	+ ns
Perceived vulnerability to malaria	+ ^b^	−^a^	+ ^b^
Discussed malaria with others	− ns	+ ^c^	−^c^
Perceived response efficacy of ITNs	− ns	+ ^c^	+ ^c^
Perceived self-efficacy to use nets	+ ^c^	+ ^c^	+ ^c^
Perceived net use as a community norm	+ ^c^	+ ^a^	+ ns

+ positive association, − negative association, *ns* not significant

^a^p < 0.05

^b^p < 0.01

^c^p < 0.001

There was no significant link with perceived response-efficacy of nets in Cameroon, but the relationship was positive in Côte d’Ivoire and Sierra Leone. The positive link found in Côte d’Ivoire and Sierra Leone echoes what Babalola et al. [32] documented in Nigeria. This study found a positive relationship between consistent net use and descriptive norms in Cameroon and Côte d’Ivoire, echoing findings from previous studies [32, 57]. However, in Sierra Leone, the association was not significant, similar to what Babalola et al. [46] and Storey et al. [39] found in their studies relating caregivers’ ideational variables to intrahousehold ITN use. Discussion of malaria with others was positively associated with consistent use in Côte d’Ivoire, echoing findings from Kilian et al. [45], and Babalola et al. [32]. In contrast, the relationship was negative in Sierra Leone and not significant in Cameroon. The lack of relationship in Cameroon is consistent with what Storey et al. [39] found for Madagascar and Nigeria in their multi-country study. The reason for the negative association between consistent net use and discussion of malaria with others in Sierra Leone is not clear.

Most household characteristics showed significant association with consistent ITN use but not consistent across all study countries. Household net coverage is the only household variable that showed a consistent positive relationship across the three study countries. This finding makes intuitive sense and is consistent with evidence from extant literature [14, 43, 66]. The only dwelling unit variable included in the estimated models—integrity of the ceiling—was negatively associated with consistent use in Côte d’Ivoire but not significant in Cameroon and Sierra Leone. The negative association in Côte d’Ivoire is consistent with what Tchinda et al. [60] found in their study in the Centre region of Cameroon where they related use of ITNs to the extent that the dwelling unit is “secured” or “unsecured” against mosquito access. They derived the measure of dwelling unit security from three variables, viz.: presence of a ceiling, presence of doors/or windows and absence of holes on walls, indicating that housing that is more “mosquito-proof” may be a factor that reduces consistent net use.

There was a positive correlation with household wealth in Cameroon, but the relationship was negative in Côte d’Ivoire and not significant in Sierra Leone. Both positive and negative patterns of association have been found in previous studies. The reason for the positive association with wealth quintile found in Cameroon was not clear; the finding was, however, consistent with what Olapeju et al. [17] found for Angola and Tanzania as well as evidence from other studies [25, 27, 35, 37]. The negative relationship found in Côte d’Ivoire echoes findings from Ricotta et al. [33], Babalola et al. [32], and Finlay et al. [41]. Certain housing characteristics and household assets that are more common among wealthier quintiles (screened windows and doors, fan, air conditioner) and that provide a sense of protection from mosquitoes may explain the negative relationship between consistent ITN use and household wealth.

Finally, the findings underscored the significant role of unmeasured variables at the EA and household levels. The residual intra household correlation, observed after adjusting for measured variables, was very significant in the three countries, indicating that net use behaviour is similar for people in the same household irrespective of household and individual characteristics. The persistence of EA-level clustering, particularly in Cameroon and Sierra Leone, echoes what Babalola et al. [32] found in Nigeria. This study does not allow for identification of the specific unmeasured variables operating at the household or EA levels that may be responsible for the observed clustering. At the household level, factors related to the attributes and net use behaviour of the head of household are possible responsible factors. At the EA level, it is possible that the factors include social norms pertaining to the use of ITN, myths and rumours about ITNs circulating within the community, local behaviour change activities to which the population is exposed, and attitudes of community leaders relative to ITN use.

The findings from this study have implications for programming, policy, and future research. For example, the substantial differences in the ideational correlates of consistent ITN use across countries indicate that regarding SBC strategies for promoting this behaviour, one approach does not fit all. Strategies will have to be adapted to country and regional context addressing the ideational variables that are found to be significant correlates of ITN use among households with nets. All the same, the consistent association of perceived self-efficacy with use of ITNs across the three countries underscores the importance of SBC efforts designed to strengthen perceived self-efficacy for ITN use. Drawing on the literature on perceived self-efficacy [67–69] such efforts should address the four sources of self-efficacy: enactive mastery, vicarious experience, verbal persuasion, and emotional state. Efforts designed to strengthen enactive mastery should encourage and empower individuals to use nets successfully for the first time, and more importantly, identify and address physical and logistic obstacles to continued use (perceived difficulty, effort required for successful performance, demotivation due to initial failure). Such efforts should also provide opportunities for discussing experience and receiving feedback. Efforts capitalizing on vicarious experience as a source of strengthening perceived self-efficacy can use modelling to communicate the message that people similar to the intended audience are overcoming barriers and successfully using an ITN. Modelling can be symbolic using the radio, television and social media to depict consistent use of an ITN by someone similar in attributes to the intended audience. Using verbal persuasion to foster perceived self-efficacy involves fostering people’s beliefs about their capability by providing constructive feedback and positive appraisals. Addressing the emotional source of perceived self-efficacy implies addressing people’s emotional response to ITN use, including fear of unintended health and social consequences.

Efforts that seek to promote positive attitudes towards ITNs, by identifying and correcting misinformation about ITNs and other ideational barriers to consistent net use, are also recommended. Strategically designed messages that emphasize the effectiveness of ITNs for malaria prevention are recommended in all three study countries. Messages about susceptibility to malaria are relevant in Cameroon and Sierra Leone where there is a positive association between this variable and consistent ITN use. The reasons for the negative relationship between perceived vulnerability to malaria and consistent ITN use observed in Côte d’Ivoire are not clear: further investigation using qualitative methods is required to better understand this unexpected finding. Findings from Cameroon and Côte d’Ivoire suggest that SBC programmes may improve consistent net use by incorporating modelling of ITN use as a community norm in behaviour change activities and messages.

The significant relationship of household net coverage with consistent ITN use observed in the three countries underscores the need for ensuring access to a sufficient number of ITNs within households as the primary facilitator of consistent ITN use. In addition to free mass and multi-channel continuous distribution of ITNs, other means of obtaining ITNs, grounded in data on willingness to pay, may be considered.

Finally, documented strong clustering of consistent use at the household level suggests that efforts that target household members as a group are relevant. Such efforts could target heads of households and key decision-makers within the household with messages that encourage them to ensure that they and other members of their household sleep under an ITN every night. Furthermore, the significant residual EA level clustering in Cameroon and Sierra Leone indicates that social mobilization strategies designed to include community perspectives and increase the use of ITNs are relevant in those countries.

The strengths of this study are numerous. The study uses a theory-based approach to identify the significant correlates of consistent ITN use. The ideation model has been used in multiple settings and found to be relevant in explaining various health behaviours. This study adds to the large body of evidence on the usefulness of the ideation model for understanding malaria-related behaviours and prioritizing interventions with potential to have a substantial impact on outcomes. The three surveys are comparable for several reasons including the fact that they were fielded in the rainy season and implemented within three years of one another thereby ensuring that there would be no huge differences in climate/rains, seismic differences in policy, or ITN distribution modalities. The use of a three-level logistic regression model with fixed effects at the individual level and random effects at the household and cluster levels is another strength of this study. Prior studies that have used multilevel modelling to assess the correlates of ITN use have generally used a 2-level approach [32, 46]. All three surveys were conducted in close collaboration with National Malaria Control Programmes in the study countries, with oversight from an advisory committee led by the Ministry of Health. Finally, findings from this study are immediately useful for in-country policy makers and programme planners in the design of policies and tailored interventions.

This study has some limitations that warrant mention. First, the data used in the analysis are cross-sectional, precluding causal inferences, and the results presented are associations. Nonetheless, the strength of the associations (both in effect size and statistical significance) indicates that the results have relevance for programmatic decision-making. Second, the data describe only households with at least one ITN and only two subnational areas within Cameroon and Sierra Leone, so findings may not be generalizable beyond these households and areas. Third, the variables in the analyses are based on self-report, thereby making the responses subject to social desirability bias. It can be argued that given the precautionary measures taken during field work to ensure that interviewees provide objective responses, such as survey questions with unprompted responses, the risk for social desirability is minimized. Last, recent net distribution campaigns may influence ideation variables and the timing of data collection was not consistently aligned with net distribution campaigns conducted in each country, although exposure to malaria SBC messages in the six months prior to the survey was controlled for in all three countries.

## Conclusions

To maximize the effectiveness of ITNs for malaria prevention, net use must be a consistent habit every night, throughout the night, most or all of the year, by all household members. This study explored the multilevel factors associated with consistent use of ITNs in three African countries among households with at least one ITN. The results show similarities and differences in the correlates of consistent use across the study areas in the three countries. In particular, the results reveal the importance of household net coverage and ideational variables for ITN use among households with at least one ITN in the study areas. Social and behaviour change programmes designed to promote consistent use of ITNs should consider using an approach based on the ideation model. Data from the MBSs provide valuable context and are useful in informing the design and implementation of social and behaviour change interventions, filling an important formative data gap.

## Supplementary Information

**Additional file 1:** **Table S1.** Results of multicollinearity test for ideational variables.

## Data Availability

The Malaria Behavior Survey datasets are available through application to the USAID open access Development Data Library at https://data.usaid.gov.
