# Supplementary material for: Ideational factors associated with consistent use of insecticide-treated nets: a multi-country, multilevel analysis
Source: Malar J. 2022 Dec 6;21:374. doi: 10.1186/s12936-022-04384-3 (PMC9724329; doi:10.1186/s12936-022-04384-3)
Supplement: Supplementary file 1 — Additional file 1: Table S1. Results of multicollinearity test for ideational variables. [file 12936_2022_4384_MOESM1_ESM.docx]

| **Supplementary Table A: Results of multicollinearity test for ideational variables** | | | |
| --- | --- | --- | --- |
| **Ideational Variables** | **Variance Inflation Factor (VIF)** | | |
|  | **Cameroon** | **Côte d’Ivoire** | **Sierra Leone** |
| Positive attitudes towards net use | 1.18 | 1.06 | 1.07 |
| Perceived response efficacy of ITNs | 1.12 | 1.05 | 1.09 |
| Perceived vulnerability to malaria | 1.05 | 1.02 | 1.13 |
| Perceived net use as a community norm | 1.09 | 1.02 | 1.03 |
| Perceived severity of malaria | 1.11 | 1.02 | 1.09 |
| Perceived self-efficacy to use nets | 1.14 | 1.01 | 1.19 |
| Discussed malaria with others | 1.09 | 1.01 | 1.10 |
| **Mean VIF** | **1.11** | **1.03** | **1.10** |
